# Regulation of blood vascular permeability in the skin

**DOI:** 10.1186/s41232-017-0042-9

**Published:** 2017-07-10

**Authors:** Sachiko Ono, Gyohei Egawa, Kenji Kabashima

**Affiliations:** 1 Department of Dermatology, Kyoto University Graduate School of Medicine, 54 Shogoin-Kawahara, Sakyo, Kyoto, 606-8507 Japan; 2 Singapore Immunology Network (SIgN) and Institute of Medical Biology, Agency for Science, Technology and Research (A*STAR), Biopolis, Singapore; 3 PRESTO, Japan Science and Technology Agency, Saitama, Japan

**Keywords:** Blood vessel, Permeability, Interendothelial junctions, Paracellular, Transcellular, Skin, Inflammation, Immunoglobulin

## Abstract

Regulation of blood vessel permeability is essential for the homeostasis of peripheral tissues. This regulation controls the trafficking of plasma contents, including water, vitamins, ions, hormones, cytokines, amyloids, lipoproteins, carrier proteins, and immunoglobulins. The properties of blood vessels vary among tissues based on their structural differences: continuous, fenestrated, or sinusoidal. These three types of blood vessels have different charge and size barrier properties. The anionic luminal glycocalyx layer on endothelial cells establishes the “charge barrier” that repels the attachment of negatively charged blood cells and plasma molecules. In contrast, the “size barrier” of blood vessels largely relies on the interendothelial junctions (IEJs) between endothelial cells, which define the paracellular permeability. As in most peripheral tissues, blood capillaries in the skin are composed of continuous and/or fenestrated blood vessels that have relatively tighter IEJs compared to those in the internal organs. Small vesicles in the capillary endothelium were discovered in the 1950s, and studies have since confirmed that blood endothelial cells transport the plasma contents by endocytosis and subsequent transcytosis and exocytosis—this process is called transcellular permeability. The permeability of blood vessels is highly variable as a result of intrinsic and extrinsic factors. It is significantly elevated upon tissue inflammations as a result of disabled IEJs and increased paracellular permeability due to inflammatory mediators. An increase in transcellular permeability during inflammation has also been postulated. Here, we provide an overview of the general properties of vascular permeability based on our recent observations of murine skin inflammation models, and we discuss its physiological significance in peripheral homeostasis.

## Background

**Fig. 1:**
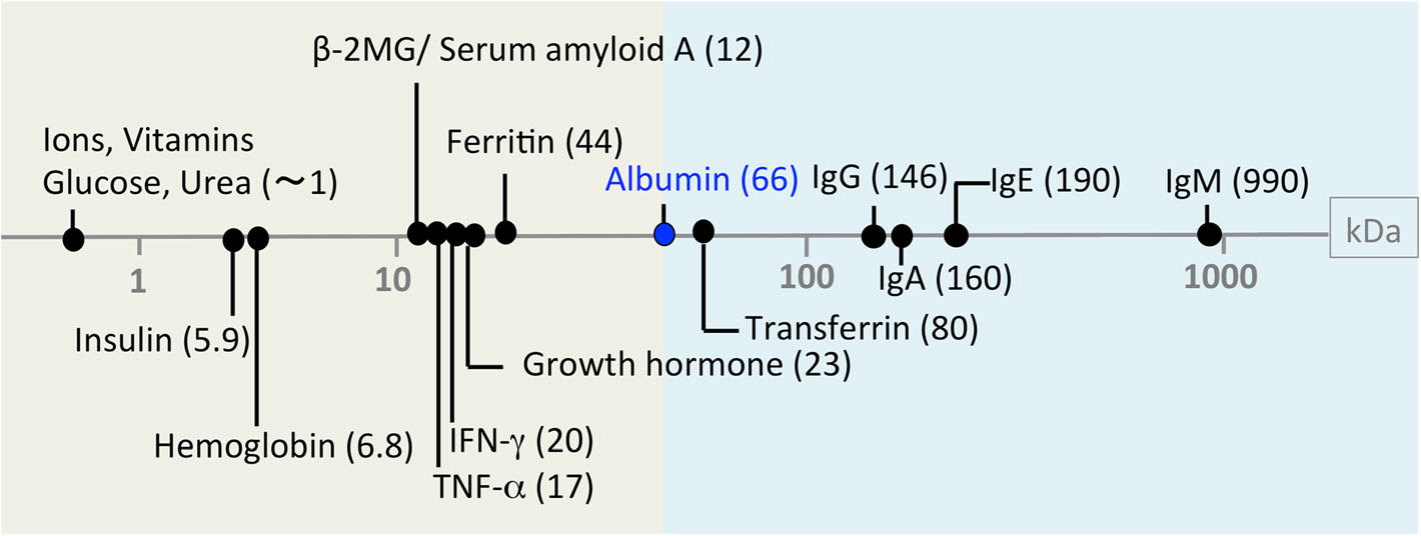
The molecular weights of representative plasma molecules. *β-2MG* beta-2 microglobulin, *IFN-γ* interferon-γ, *TNF-α* tumor necrosis-α (Modification from a figure in [14]). The background colors discriminate plasma molecules that may (*gray*) or may not (*blue*) extravasate via paracellular pathway of the cutaneous blood vessels

Blood vessels, especially those of microvessels, serve as a semipermeable barrier between blood contents and the tissue, which is much more permeable than epithelial systems. Acting as canals, blood vessels carry cargos with different sizes and charges in plasma to their proper destinations (Fig. 1).

**Fig. 2:**
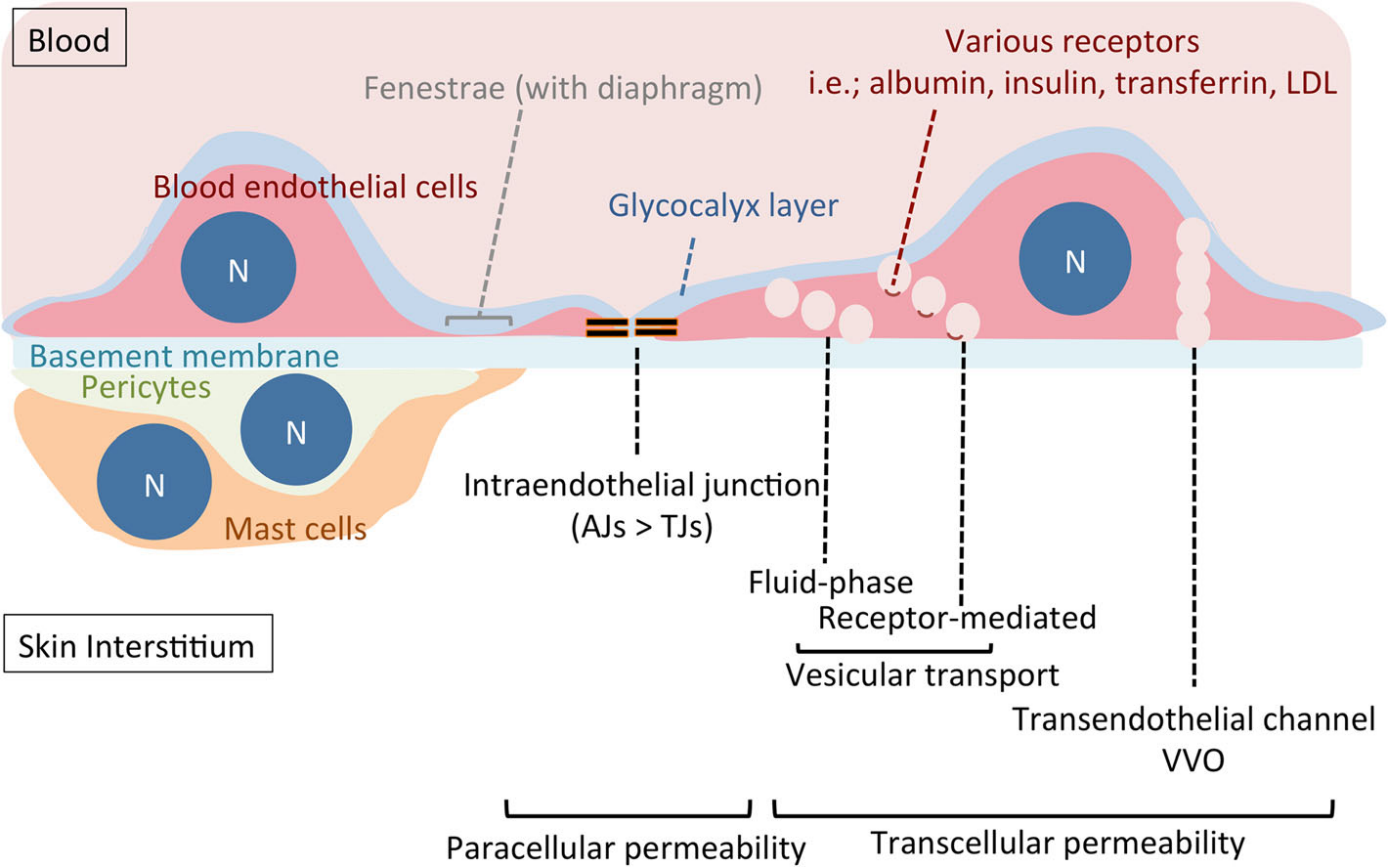
Integrity of blood vessels in the skin. *N* nucleus, *AJs* adherens junction, *TJs* tight junction, *VVO* vesiculo-vacuolar organelle, *LDL* low-density lipoprotein

The permeability of blood vessels is composed of two distinct barriers: the charge barrier and the size barrier (reviewed in [1–3]). The luminal glycocalyx layer on endothelial cells establishes the anionic “charge barrier,” with some additional roles have been postulated to date (discussed later). The paracellular permeability between the interendothelial junctions (IEJs) is often responsible for the size barrier, which is regulated by the presence or absence of adherens junctions (AJs) and/or tight junctions (TJs) in the IEJs (reviewed in [1, 4]). However, IEJs are not solely responsible for defining the size barrier; there appears to be a large contribution of basement membranes, fenestrae, and diaphragms [3] (Table 1). In addition to endothelial organization, non-cellular and cellular components surrounding blood endothelial cells, the extracellular matrix ([5], reviewed in [6]), pericytes [7], and immune cells such as perivascular mast cells, may participate in regulating the permeability of blood vessels [8] (Fig. 2). Furthermore, in terms of vesicular transportation through endothelial cells, the transcellular pathway may dominate the paracellular pathway in determining the vascular permeability of selective molecules, especially in vessels with tight IEJs.

**Table 1:** Types of blood vessels in various organs with different permeability

A. Charge barrier [17–23]					
Glycocalyx layer	Anionic mesh-like layer with regular spacing of <20 nm for continuous and fenestrated vessels (irregularly found on sinusoidal vessels), on both the surface of IEJ clefts and endothelial cells.				
B. Size barrier (reviewed in [2])					
Types of blood vessels		Types of endothelial cells	Interendothelial junctions (IEJs)	Representative organs	Estimated upper limit for paracellular transportation [4]
Continuous(non-fenestrated)	Continuous basement membrane	No fenestrae	Tight junctions and adherens junctions	Retina [2] brain, spinal cord [66] thymus [67]	Determined by IEJs (TJs) <1 nm
			Adherens junctions with limited contribution of tight junctions	skin [12, 13] muscle, heart [68, 69] adipose tissue [70] lung [71, 72]	Determined by IEJs (AJs) <5 nm
Fenestrated		Fenestrated (with diaphragm)		skin [12, 13] exocrine glands [73] kidney (peritubular) [74] endocrine glands [73, 75, 76] intestinal mucosa [77, 78] lymph node [79, 80]	Determined by diaphragm <6–12 nm [81]
		Fenestrated (open pores without diaphragm)		Kidney (glomerulus) [82, 83]	Determined by glycocalyx <15 nm [2, 19]
Sinusoidal (discontinuous)	Discontinuous basement membrane	Fenestrated (with and/or without diaphragm)		Liver [84–86] spleen [87]	<50–280 nm, largely differ among species <3–5 μm

Here, we provide an overview of the current knowledge of the permeability of blood vessels. We then cut into the dynamic regulation of blood vascular permeability especially upon inflammation. We also focus on the extravasation of immunoglobulins (Igs), the representative macromolecules in plasma, to the skin, because they may be essential for the homeostasis of cutaneous immune systems not only in terms of host protection but also for the pathogenesis of allergic and autoimmune skin disorders.

## Types of blood vessels and their size barriers determine paracellular permeability

The human body has three types of blood vessels based on their structural differences: continuous (non-fenestrated), fenestrated, and sinusoidal (reviewed in [2]). In brief, blood vessels can first be classified into sinusoidal (discontinuous) or non-sinusoidal by the presence or absence of continuous basement membranes beneath endothelial cells. Non-sinusoidal blood vessels can be termed as continuous blood vessels in a broad sense and can be further classified into fenestrated and non-fenestrated (continuous blood vessels in a narrow sense), based on their endothelial types with or without fenestrations. Fenestrated blood vessels can further be sub-classified by the existence of a diaphragm [9] (Table 1).

IEJs, the structures connecting adjacent blood endothelial cells, are composed of AJs and TJs. AJs are composed of vascular endothelial (VE)-cadherin complexes with catenin; and TJs are composed of claudins, occludins, and junctional adhesion molecules [1, 4, 10]. In human umbilical vein endothelial cells, TJs represent only approximately 20% of the total junctional complexes [11]. Therefore, it is generally accepted that IEJs are primary established by AJs in most peripheral blood vessels (reviewed in [4]). In specific continuous vessels, blood endothelial cells are much more firmly adhered to each other with enriched TJs to serve as specialized interfaces such as the blood-brain barrier or the blood-retinal barrier, bringing about low accessibility of plasma contents to these tissues.

The blood vessels in the skin are reportedly composed of continuous (non-fenestrated) and fenestrated blood vessels [12, 13], limiting passive diffusion of albumin, which has the molecular size of 66 kDa (approximately 7 nm in a diameter), and of dextrans larger than 70 kDa (as discussed later) [14]. This is consistent with the previous studies on other continuous vessels [2, 15]. Taken together, cutaneous blood vessels may act as the size barrier around 70 kDa, presumably allowing the passive diffusion of small molecules, including ions, glucose, urea, amino acids, insulin, cytokines, amyloids, and some hormones via the paracellular pathway in the steady state but not of albumin, transferrin, and Igs (Fig. 1). Of note, the size barrier only reflects one aspect of overall vascular permeability because the extravasation of each plasma molecule may be induced by the transcellular and paracellular permeability with variable dependency (Fig. 2).

## The charge barrier

Glycocalyx is a negatively charged continuous coat of proteoglycans, glycosaminoglycans, and absorbed plasma proteins, on the luminal surface of blood endothelial cells [4, 16, 17] (Fig. 2). Its thickness has been reported to range between 20 and 3000 nm depending on the detection method, vessel types, and the tissues [17–20]. Glycocalyx acts as a primary charge barrier for the transportation of plasma molecules. Several studies using enzymatic procedures that induce shedding or disruption of the glycocalyx layer or neutralize its negative charge have demonstrated the increased vascular permeability to water without affecting the IEJs [20–23].

Glycocalyx can also act as the primary size barrier in fenestrated blood vessels. In these vessels, the diameter of endothelial fenestrations is around 60 nm irrespective of the presence of a diaphragm, but the physiologically estimated upper limit of the size barrier is smaller than 15 nm [2] (Table 1). It is assumed that this discrepancy may be due to glycolcalyx occupying the fenestrations [18]. These observations lead to the “fiber matrix” theory, the idea that glycocalyx’s fiber mesh-like structure with regular spacing of 20 nm may regulate vascular permeability [19]. Glycocalyx may modulate the permeability of plasma molecules, and in turn, plasma proteins can be an intrinsic part of glycocalyx [3, 24]. In this context, it is interesting to consider that plasma molecules can indirectly regulate the vascular permeability of other plasma molecules. Glycolcalyx can also sense a fluid shear stress and induce endothelial nitric oxide synthesis within endothelial cells to stabilize the barrier function of blood vessels [25].

## The drastic increase in vascular permeability upon various cutaneous inflammations

Both the size and the charge barriers of blood vessels are largely affected by the physiological state of the surrounding tissue interstitium. These changes in permeability were conventionally assessed by an in vitro transwell assay system that measured the flux of variable molecules through the endothelial cell monolayer cultured in transwell chambers under various stimulus agents [26–29]. Despite the utility of the assay, it has frequently been pointed out that this assay system might not reconstitute the actual vascular integrity and permeability in vivo (discussed in [27]). Alternatively, the Miles assay has been widely used to assess vascular permeability in mice [30]. Intravenously administered tracers (such as Evan’s blue) bind to albumin, and the accumulation of the tracer in the skin is evaluated after the local administration of stimulants to evoke vascular hyperpermeability. The Miles assay is useful in evaluating gross changes in vascular permeability in vivo but lacks anatomical information, i.e., the site of hyperpermeability in the net of blood vessels or the interaction of endothelial cells with perivascular cells. Furthermore, the subtle extravasation of tracers in the steady state is under the detection limit in the Miles assay.

In addition to these conventional methods, a new intravital evaluation system for vascular permeability in mice using two-photon microscopy has revealed in a more detailed manner how the blood vascular permeability is dynamically regulated in vivo in the skin [14]. By the intravenous administration of different sizes of fluorescein-conjugated dextrans (20 to 2000 kDa), it was clearly visualized that the passive diffusion, which may reflect the paracellular transportation, occurs only when dextrans are smaller than 70 kDa. When fluorescein-conjugated bovine albumin (molecule size 66 kDa) was administered intravenously, the majority seemed to be retained in the blood. A gradual extravasation was, however, observed within 1 h after an injection of albumin but not for 70 kDa dextrans. This may reflect the different regulation of the transcellular transportation of albumin and dextran with similar size. The same in vivo system also clarified the site of vascular hyperpermeability induced in both type I and type IV allergic cutaneous inflammation. Upon inflammation, the size limitation for plasma molecules was abolished, allowing the immediate leakage of up to 2000 kDa dextrans to the skin interstitium. This leakage was selectively induced in the postcapillary venules. This corresponded to the previous assumption that postcapillary venules are the specific site of vascular leakage in inflammation. The physiological barrier of the postcapillary venules seems intrinsically sensitive and vulnerable to inflammation, due to abundant receptors for chemical mediators such as histamine and bradykinin [31, 32], less-abundant TJs [33], and low coverage rate by pericytes of these vessels [34]. Numerous chemical mediators, which are released upon inflammation, can lead to diminishment of AJs and the contraction of blood endothelial cells that lead to the formation of IEJ gaps in postcapillary venules. The molecular detail of underlying mechanism for the dysregulation of paracellular permeability is discussed in other reviews [4]. In addition to vascular leakage, postcapillary venules can also serve as the specific site of leukocyte infiltration and inflammatory cell gathering, which is essential for immune responses in the skin [35–38].

As discussed later, the transcellular pathway might play a central role in the extravasation of plasma macromolecules in the steady state. It is of note that the increase in the transcellular transportation of albumin due to increased caveolae function has also been demonstrated in inflammation [39]. Furthermore, the regularity of glycocalyx is disrupted upon inflammation, resulting in irregular thickened layers and gaps between them. Clustering of glycocalyx induced by inflammation can also activate intracellular signals and provoke cytoskeletal reorganization that leads to barrier dysfunction. This change in glycocalyx structures may also contribute to the elevation of permeability, although this appears to be ignored in recent studies. Overall, the changes in the paracellular permeability, the transcellular permeability, and the charge barrier can all participate in gross increase in vascular permeability upon inflammation.

## The increase in immunoglobulin G extravasation to the skin upon inflammation

As mentioned in the previous sections, the drastic increase in vascular permeability might allow the extravasation of plasma contents, including macromolecules. Among them, here, we focus on the regulation of IgG and IgE extravasation in the skin because they may play important roles in the terms of protective and pathological immune reactions in the skin.

Historically, IgG kinetics has mostly been studied in the intestinal epithelia or the placenta in view of maternal-to-neonatal/fetal IgG passage. The necessity of the neonatal Fcγ receptor in epithelial cells and trophoblasts has well been established; however, few studies have examined IgG kinetics at the blood vessel walls [40–45]. The molecular weight of IgG is approximately 150 kDa (Fig. 1). It was thus presumed that the extravasation of IgGs is tightly regulated in the steady state.

Recent observation using a murine pemphigus model, which is a representative model for autoantibody-related disorders in the skin, revealed that variable local inflammation, such as ultraviolet B irradiation or the topical application of irritants to the skin, enhanced autoantibody deposition in the skin [36]. This increase in autoantibody deposition in the skin leads to exacerbated skin manifestation in the murine pemphigus model. The human body is frequently exposed to external stimuli such as frictions, heat, and the sunlight, which can elicit minor local inflammation. Therefore, IgG distribution in the periphery might be largely influenced by external circumstances. Indeed, it is well known that IgG deposition in the epidermal basement membrane is more frequently detected in sun-exposed sites in patients with systemic lupus erythematosus. In view of host protection, enhanced IgG recruitment into the inflammatory site would be important for neutralization of invading pathogens.

Despite the strict regulation, constitutive IgG extravasation to the tissue parenchyma in the steady state appeared to exist [36], and the same observation was made for albumin. This homeostatic extravasation of plasma macromolecules may rely on transcellular permeability (Table 2).

**Table 2:** Transportation of plasma contents in the steady state

Routes	Molecules		
Paracellular pathway	Water molecules <3 nm molecular radius (i.e., urea, amino acids, glucose, ions)		
Transcellular pathway	Aquaporin channels		Water (up to 40% of total hydraulic pathway)
	Fluid-phase	Caveolae	Albumin [27, 46] intact native, acetylated and oxidized LDL [88, 89] IgG [44] transferrin and iron [90]
		Undetermined	IgG (bound to FcRn in endosomes after fluid-phase endocytosis [40, 41, 43, 49])
	Receptor-mediated	Caveolae	Albumin (via gp60 receptor) [50–52] insulin (via unknown receptor) [49]
		Clathrin	Insulin [91] transferrin and iron (via transferrin receptor [92, 93]) gonadotrophin (via gonadotropin receptor [94])
		Undetermined carrier vesicle	IgG (via FcRn or FcγR2b [45, 95, 96]) LDL (via LDL receptor [97]) insulin (via insulin receptor [98])
	Transendothelial channels		
	Vesiculo-vacuolar organelles		
Direct probing by non-endothelial cells over blood vessels	IgE (via FcεRI by mast cells) [58]		

## Importance of transcellular permeability

Conventionally, it is considered that there are two different types of transcellular pathway: receptor-mediated transcytosis and non receptor-mediated bulk-phase transcytosis (often called “fluid-phase” transcytosis) [27, 46] (Fig. 2). In this review, we do not discuss the transendothelial channels or vesiculo-vacuolar organelles [47, 48]. Plasma molecules those are smaller than the size barrier of the blood vessels (<70 kDa), like insulin, might be able to extravasate in both paracellular and transcellular pathways. However, the transporting efficiency is reportedly much higher in paracellular transportation [49, 50]. Plasma macromolecules that are larger than the size barrier of the blood vessels (>70 kDa) might extravasate by either fluid-phase or receptor-mediated transcytosis; however, its balance in vivo for most macromolecules has not been elucidated.

The transcellular permeability of albumin has extensively been studied and found to be largely dependent on the receptor-mediated transcytosis via gp60 in caveolae [51–53]. Even for albumin, to what extent fluid-phase transcytosis contributes to the overall albumin extravasation remains undefined. Furthermore, in fluid-phase transcytosis, it is believed that the selectivity of molecules might exist, due to their size and charge. Collectively, the mechanism of transcellular transportation remains to be elucidated for most plasma molecules. The proposed routes for the extravasation of plasma molecules are shown in Table 2.

In epithelial cells, the transcellular pathway is initiated by endocytosis [27]. Therefore, it might also be important to define the way of endocytosis of each molecule to understand the mechanism of transcytosis in blood endothelial cells. Endocytosis can define the destinations of the contents, i.e., to lysosomal degradation, to recycling, or to the transcellular pathway [40–42, 54, 55] (discussed in [51]). Various forms of endocytosis by eukaryotic cells have been found to date, including phagocytosis, macropinocytosis, clathrin-mediated endocytosis, clathrin-independent caveolae-mediated endocytosis, and newly defined clathrin-independent non-caveolar endocytosis [56, 57]. Because caveolae are abundantly observed in blood endothelial cells [50], it is sometimes oversimply stated that both fluid-phase transcytosis and receptor-mediated transcytosis is mediated by caveolae. However, the abundance of caveolae can vary widely among blood vessels in different tissues [27, 46]. Some studies have suggested the possibility of endocytic pathways other than caveolae in blood endothelial cells (Table 2), but we believe that the actual contribution of various endocytic vesicles on transcellular transportation should be more rigorously explored. In addition to investigating the transcellular route for each macromolecule, their relation to intracellular membrane organelles, such as early endosomes, sorting endosomes, or lysosomes, is also essential in order to understand their final destination. Transcellular permeability is a key issue that requires further research to improve our understanding of vascular homeostasis.

## Another unique style of molecular extravasation in the skin—immunoglobulin E

A unique extravasation mechanism of IgE in the skin has recently been demonstrated using an in vivo imaging technique [58]. Mast cells are abundantly located in the skin along the blood vessels [8]. Mast cells are best known as the effector cells of IgE-mediated allergic responses, such as allergic dermatitis and urticaria. Under crosslinking of high-affinity IgE receptors on their surface by specific antigens, mast cells are activated and release proinflammatory molecules, including histamine, leading to vascular hyperpermeability. Intriguingly, recent studies have demonstrated that perivascular mast cells capture blood-circulating IgE by extending their processes across the vessel wall in the steady state [58]. Because the plasma concentration of IgE is significantly lower compared to other Igs and proteins, the way in which mast cells probe and capture IgE by their surface high-affinity IgE receptor (FcεRI) appears to be strategic.

No studies have properly assessed the transcellular transportion of IgE, IgA, and IgM via blood endothelial cells. In addition, low-affinity IgE receptors (CD23) or polymeric Ig receptors in epithelial cells have been reported responsible for the transcellular transportation of IgE or IgA and IgM [59–63]. Discriminating the difference between endothelial systems and epithelial systems would reveal the characteristic nature of the blood-tissue interface.

## Conclusions

The regulation of blood vessel permeability is important for tissue homeostasis and has attracted the attention of vascular biologists for decades. Considering that nanoparticles [64], antibody-based biologics, or immune checkpoint inhibitors [65] are globally accepted as promising therapeutic tools for autoimmune disorders and various cancers, the basic insight into the kinetics of micro- and macromolecules at the blood-tissue interface would provide a practical clinical information. By employing accumulated knowledge and well-established conventional methods, the in vivo techniques introduced in this review to finely evaluate blood vascular permeability would enable an enhanced understanding of this physical process.

## Abbreviations

AJs: Adherens junctions
IEJs: Interendothelial junctions
Ig: Immunoglobulin
TJs: Tight junctions
